# Prevalence and Exposure Assessment of *Alternaria* Toxins in Zhejiang Province, China

**DOI:** 10.3390/foods14193298

**Published:** 2025-09-23

**Authors:** Zijie Lu, Ronghua Zhang, Pinggu Wu, Dong Zhao, Jiang Chen, Xiaodong Pan, Jikai Wang, Hexiang Zhang, Xiaojuan Qi, Shufeng Ye, Biao Zhou

**Affiliations:** 1 School of Public Health, Hangzhou Medical College, Hangzhou 310013, China; 130232023409@hmc.edu.cn; 2 Zhejiang Provincial Center for Disease Control and Prevention, Hangzhou 310051, China; rhzhang@cdc.zj.cn (R.Z.); pgwu@cdc.zj.cn (P.W.); dzhao@cdc.zj.cn (D.Z.); jchen@cdc.zj.cn (J.C.); xdpan@cdc.zj.cn (X.P.); jkwang@cdc.zj.cn (J.W.); hxzhang@cdc.zj.cn (H.Z.); xjqi@cdc.zj.cn (X.Q.); 17867970961@163.com (S.Y.); 3 School of Public Health, Ningbo University, Ningbo 315211, China

**Keywords:** *Alternaria* toxins, pollution status, dietary exposure, risk assessment

## Abstract

This study aimed to assess the prevalence of four *Alternaria* toxins (alternariol [AOH], alternariol monomethyl ether [AME], tenuazonic acid [TeA], and tentoxin [TEN]) in various foods and assess the risk of *Alternaria*-toxin exposure in Zhejiang Province, China. A total of 325 samples were collected in this study, and at least one type of *Alternaria* toxin was detected in 53.85% of the samples. Wheat flour had a high detection rate of 97.41%, and TeA was the most prevalent compound in terms of concentration and detection rate. Assessment of *Alternaria* toxins using the threshold of toxicological concern (TTC) method showed that the majority of the population had a low exposure risk. Population-wide dietary exposure assessment suggested a potential health risk for some residents with 95th percentile (P95) assessment values 0.0038, 0.0128, and 0.0047 µg/kg b.w. for AOH from wheat flour and AOH and AME from Coix rice, respectively, exceeding the TTC value of 0.0025 µg/kg b.w. Probabilistic assessment showed that the mean exposure of children aged ≤6 years to AOH via wheat flour for P92 and of those aged 7–12 years for P93 were both 0.0025 µg/kg b.w. Exposures to TeA and TEN were within the acceptable limits (below the TTC value of 1.5 µg/kg b.w.). Age-group probabilistic and point assessments indicated that children aged ≤6 and 7–12 years are at higher exposure risk. This study provides a useful reference for developing limiting values and legislation for *Alternaria* toxins in food.

## 1. Introduction

*Alternaria* species are saprophytic fungi, usually found in soil or decaying plant tissues, which can cause various crop diseases or post-harvest rots and are widely distributed in nature. More than 70 secondary metabolites of *Alternaria* have been reported worldwide; five categories of *Alternaria* are reported based on their chemical structure, including perylene quinone derivatives, dibenzopyrone derivatives, tetramic acid derivatives, aminopentol esters, and miscellaneous structures, of which the most common *Alternaria* toxins are alternariol (AOH), alternariol monomethyl ether (AME), tenuazonic acid (TeA), tentoxin (TEN), and altenuene (ALT) [1,2,3,4].

*Alternaria* species are highly environmentally adaptable and stable. They can survive at 4 °C and have an acceptable pH range of 2.7–8.0 [2,5,6]. The toxins they produce are not easily removed during food processing [2,4]. Both AOH and AME are lipophilic and soluble in most organic solvents [6,7]. They remain stable at 100 °C; although their levels decrease after 60 min at 121 °C, the treated food can cause kidney damage in rats [8]. Owing to their characteristics, toxins tend to accumulate in food, and the intake of contaminated food has the potential to cause harm to the human body.

Environmental conditions, crop species, processing methods, and other factors affect crop contamination with *Alternaria* toxins. *Alternaria* species grow widely in humid and semi-arid areas [2], with an optimum growth temperature of 22–30 °C, higher water activity (a_w_), and humidity, promoting its production [1,9]. Cereals, fruits, and vegetables are foods at high risk of contamination with *Alternaria* toxins, and the abundance of water and nutrients in fruits and vegetables provides favorable conditions for *Alternaria* toxin production [2,10,11]. Dehydration and concentration of fruit products can lead to increased *Alternaria* concentrations, whereas hulling or dry roasting of cereal products can reduce contamination and concentrations [10,12].

The metabolism and excretion of *Alternaria* toxins in the body may also affect their toxicity. AOH and AME produce hydroxylated metabolites in phase I metabolism that are less damaging to cellular DNA than the metabolites themselves. Hydroxylation reduces their ability to bind to the estrogen receptor; most phase I metabolites further form conjugates with glucuronic acid and sulfuric acid [13,14,15]. To our knowledge, no studies have yet examined the metabolic processes of TeA and TEN in humans. The bioavailability of AOH in rats after oral administration is low, with blood concentrations not exceeding 0.06% of the total dose; metabolites can be detected in the urine, and the remaining 90% of unabsorbed AOH is excreted in the feces [6,7,16]. The absorption rate of AME in the gastrointestinal tract is very low; 84.6% of the administered dose of AME in rats will be excreted after 3 days, and the residual portion of the administered dose is mainly concentrated in the fat and intestines, whereas 30% of TEN will be excreted in the feces [15,17]. TeA is highly bioavailable in the body, with approximately 87–93% excreted in the urine after 24 h [18].

*Alternaria* toxins exert cytotoxic, immunotoxic, genotoxic, and reproductive and developmental effects. AOH is primarily metabolized in the liver [7,19], and its cytotoxicity is mainly manifested by interfering with the cell cycle, inhibiting cell proliferation, and inducing apoptosis [20,21]. They also suppress the immune response in an inflammatory cellular environment, making the body more susceptible to diseases and producing immunotoxicity [10,22]. AOH and AME are genotoxic and mutagenic, induce mutations at gene loci, and cause DNA strand breaks [22,23]. AOH is weakly estrogenic, and both AOH and AME reduce the synthesis of the steroid hormone progesterone, which affects reproductive performance in mammals [21,24]. TeA is more acutely toxic than AME and AOH. In acute toxicity studies of TeA, the median lethal doses (LD50) in male and female mice were 162 and 115 mg/kg, respectively, by intravenous injection, and the LD50 for oral administration was 186 and 81 mg/kg b.w., respectively [2,25]. In a 33-day short-term trial in monkeys, no adverse effects were observed at doses of 22.4 and 48.8 mg/kg b.w. TeA administered at 89.6 mg/kg b.w. caused vomiting, bloody stools, and gastrointestinal hemorrhage [2,26]. TeA has also been associated with a blood disorder called “Onyalai” and a high incidence of esophageal cancer in Lin County, China [1,27]. TEN affects plant seedling development, but its risk to humans remains unclear [10,28].

*Alternaria* toxins are found in nuts, vegetables, fruits, dried fruit products, and some alcoholic beverages, particularly in tomatoes and grains [29,30,31,32]. Spanish berry juice is 73% and 67% positive for AOH and AME, with the highest concentrations being 85 ng/mL and 308 ng/mL, respectively [33]. In Germany, the concentrations of TeA in sunflower seeds, tomato products, and bakery products were 375.3, 193.7, and 133.8 μg/kg, respectively, which were higher than those of AOH, AME, and TEN [34]. The highest concentration of TeA detected in beer was 16.5 μg/L [35]. AOH and AME were found at a dose of 63 and 12 ng/g, respectively, in Canadian cereal products and 4.4 and 9.0 ng/g, respectively, in infant cereal products [36]. The detection rates of AME, AOH, TEN, and TeA in foods in the Netherlands were 5%, 7%, 15% and 22%, respectively. TeA contamination was detected in dried figs at up to 2345 μg/kg [37]. In China, the detection rates of AOH, AME, TeA, and TEN in wheat and its products were 49.2% (mean, 0.75 μg/kg), 86.4% (0.57 μg/kg), 97.7% (27.2 μg/kg), and 96% (4.78 μg/kg), respectively [38]. Furthermore, metabolomics studies using advanced UHPLC-Q-TOF-MS methods have also helped expand the scope of the study by showing Alternaria metabolites in situ in fruit matrices [39,40,41].

Although previous studies have investigated *Alternaria* toxins in various food items, limited toxicological data are available on its role as an emerging mycotoxin, and the tolerable daily intake (TDI) for *Alternaria* toxins has not yet been established. Based on their potential genotoxicity, the European Food Safety Authority (EFSA) has adopted a threshold of toxicological concern (TTC) for the assessment of the effects of *Alternaria* toxins on humans [3]. As AME and AOH are genotoxic, the TTC value was set at 0.0025 µg/kg b.w.; no genotoxicity was detected for TeA and TEN, and their TTC values were set at 1.5 μg/kg b.w. [3,30]. The EFSA results showed that dietary exposures to AOH and AME exceeded the TTC values, exposure levels of TeA and TEN were unlikely to have health effects, and children were at higher risk of exposure due to higher exposures per kg [2,3]. A Chinese study also resulted in the same conclusion [42]. The average exposure of Chinese infants and young children to AME, AOH, and TeA through the consumption of cereal-based infant supplements is 0.002, 0.004, and 0.12 μg/kg b.w., respectively [43]. In Korea, the average dietary exposure to AOH and AME through marketed food is 0.0118–0.0506 ng/kg b.w. (lower bound [LB]-upper bound [UB]) and 0.0101–0.0378 ng/kg b.w., respectively [44]. To the best of our knowledge, there are few risk evaluations on *Alternaria* toxins, mainly for food products such as tomato and its products and fruit juices, and most of them have been conducted using point assessment models [10,45,46,47]. Although point assessment is simple and easy to use, it neglects to observe individual variability owing to different consumption rates and levels of chemical residue concentrations in food, resulting in crude and conservative results [48]. Probabilistic assessment can avoid these shortcomings.

Located on the southeast coast of China, Zhejiang Province is among the nation’s most economically developed regions. To the best of our knowledge, similar surveys have been carried out in other provinces, while no studies have been conducted on the current status of *Alternaria* toxin contamination in food or dietary exposure assessment of *Alternaria* toxins in the population of Zhejiang Province. The objectives of this study were: (i) To determine the levels of four *Alternaria* toxins (AOH, AME, TeA, and TEN) in local cereals and fruits; (ii) To estimate the exposure of consumers of different age groups by using a point assessment method combining the contaminant concentration and consumption; and (iii) to conduct a probabilistic assessment to calculate the exposure of the population to AOH through wheat flour intake, enhancing the precision of the results.

## 2. Materials and Methods

### 2.1. Sample Collection

Fruit samples were collected in 2019, and cereals and their products in 2022, respectively, by trained investigators from family farms, local retailers, supermarkets, and e-commerce platforms, in accordance with the sample collection requirements, with at least two samples of 500 g each. Sample testing was conducted by a technical monitoring agency or external laboratory as soon as possible after collection, under near-existing storage conditions. The sample size was calculated as follows [49]:

(1) $$N=\frac{Z^{2}\;\times \;\left[P\times \left(1-P\right)]\right.}{e^{2}}$$

Here, *N* denotes the sample size, and *Z* represents the 95% confidence interval (*Z* = 1.96), *P* is the expected contamination rate of the sample (*P* = 0.5), and e is the margin of error (e = 10%). A total of 325 samples were collected, including wheat flour (n = 116), maize and its products (n = 75; maize kernels and flour), Coix rice (n = 34), and fresh fruits (n = 100; grapes, apples, Mandarin oranges, prunes, and loquats). Food groups that are more commonly consumed by the population in Zhejiang Province were selected.

### 2.2. Chemical Reagents

AME, AOH, TeA, and TEN standards were obtained from Romer Labs (Union City, MO, USA). Methanol and acetonitrile were purchased from Fisher Scientific (Waltham, MA, USA). Analytically pure ammonium bicarbonate and formic acid (purity ≥ 95) were purchased from Fluka (Steinheim, Germany). Pure water was obtained using a Millipore Milli-Q apparatus (Millipore, Bedford, MA, USA).

### 2.3. Sample Preparation and Analysis

The methodology and operational procedures for the detection of *Alternaria* toxins in food were in strict accordance with the Workbook for National Food Contamination and Risk Factor Monitoring, developed by China’s National Center for Food Safety Risk Assessment (CFSA).

Briefly, 5 ± 0.001 g of sample was weighed, and 25 mL of acetonitrile-water-methanol (45:45:10, *v*/*v*/*v*) was added to it followed by vortexing for 10 s and extracting by shaking on a high-speed vortex oscillator for 15 min. This sample was then centrifuged (10,000 rpm, 10 min, and 4 °C), and 5 mL of the supernatant was taken. The supernatant was eluted with 5 mL of methanol and acetonitrile solution and evaporated using nitrogen blown over a 45 °C water bath. The supernatant was then centrifuged and analyzed using Liquid Chromatography-Tandem Mass Spectrometry (Shimadzu, Kyoto, Japan). The samples were analyzed using a Shimadzu LC-30AD (Shimadzu) and an AB 6500 triple quadrupole mass spectrometer equipped with an electrospray ionization source (AB Sciex Inc., Framingham, MA, USA). A Waters BEH C18 (2.1 mm × 100 mm, 1.7 μm) analytical column (Waters Corporation, Milford, MA, USA) was used. The mobile phase consisted of 1.0 mmol/L ammonium bicarbonate solution (solvent A) and methanol (solvent B); the flow rate was 0.25 mL/min, and the sample volume was 10 μL. The column temperature was maintained at 40 °C.

### 2.4. Method Validation and Quality Control

This study was conducted at the NHC Specialty Laboratory of Food Safety Risk Assessment. The parameters considered, including the linearity, sensitivity, accuracy, and precision of the target analyte, were validated. All the samplers, inspectors, and managers involved in this study had relevant technical experience and passed the quality management training assessment. The limit of detection (LOD) was based on a signal-to-noise ratio (S/N) of 3:1. The LOD ranged from 0.3 to 3 µg/kg^−1^ depending on the toxins, and the specific values for LOD and LOQ are shown in Table S1. To perform the exposure assessment and risk characterization, 0 (LB), 1/2 LOD (medium bound [MB]), and LOD (UB) were used instead of non-detected values [49].

### 2.5. Data on Food Consumption

Consumption data was extracted from the consumption survey conducted in Zhejiang Province between 2015 and 2017. The data were internally collected by the Zhejiang Provincial Center for Disease Control and Prevention and have not been published. Permanent residents who had been in the area for at least 6 months participated in the questionnaire, and an informed consent form was signed by each survey respondent. Household surveys were conducted in 10 counties (cities) across the province by trained and qualified enumerators to count the frequency and quantity of food consumed by residents, as well as basic information such as age, ethnicity, education level, and occupation, all personal data were treated as confidential. Owing to the lack of consumption of Coix rice, the average, P50, and P95 consumption with reference to the relevant literature were 29.82, 20, and 88.85 g/day, respectively, and the body weight was calculated with a baseline of 60 kg [50].

Participants were assigned to one of five age cohorts: children (≤6 years), older children (7–12 years), adolescents (13–17 years), adults (18–59 years), and older adults (≥60 years) [51].

### 2.6. Dietary Exposure Assessment Methods

#### 2.6.1. Point Assessment

A deterministic method was employed to evaluate the dietary exposure of the population of Zhejiang Province, which was calculated by integrating data on food consumption with contaminant concentration levels across various food commodities. The formula for the point assessment is as follows [52]:

(2) $$PDI=\frac{Contamination\;of\;concentrations\;in\;food\;(µg/kg)\;\times Consumption\;of\;food\;{(g/d}^{-1})}{Body\;weight\;(kg)}\times 10^{-3}$$

Here, *PDI* is the probable daily intake. Three assessment models were used: the mean, P50, and P95 pollutant concentrations and consumptions. In the population-wide exposure assessment, MB values were used for pollutant concentrations, and the age-grouped exposure assessment combined LB, MB, and UB values for assessing pollutant concentrations. Multiple models were used to assess the exposure and risk for most of the population, as well as for extreme cases.

#### 2.6.2. Hazard Quotient (HQ)

*HQ* is the ratio of potential exposure to a hazardous substance to the TDI or Acute Reference Dose, assessing whether acceptable levels are exceeded [53]. Due to the lack of toxicity data, the *TTC* proposed by EFSA was used instead of the TDI to assess the potential dietary health risks to consumers. The following formula is used for the calculation [30]:

(3) $$HQ=\frac{EDI}{TTC}\times 100\%$$

Population-wide dietary exposure to the four toxins was assessed using the *HQ* method. In general, an *HQ* value < 100% indicates that the exposure is unlikely to cause health effects. Conversely, if the HI value exceeds 100%, adverse effects are possible.

#### 2.6.3. Probabilistic Risk Assessment

We used R to implement a probabilistic assessment framework combining Monte Carlo simulation and bootstrap sampling to quantify uncertainty and variability in population exposure assessments. The steps were as follows. Consumption data were discrete, uniformly distributed, such that each sample had an equal probability of being drawn. Overall, 1000 bootstrap samples were obtained from the raw dietary survey data and combined with individual consumption and age-specific weight data; further, 10,000 Monte Carlo simulations were performed for each bootstrap sample to calculate daily exposure. For each bootstrap sample, the P91–96 percentile was calculated, and a 90% confidence interval was constructed from the results to reflect sampling uncertainty.

### 2.7. Statistical Analysis

The IBM SPSS software (version 25.0; IBM Corp., Armonk, NY, USA) and R 4.3.3 were used for analyzing the data. Means, medians, and 95th percentiles are used to display quantitative data, and count data are expressed as ratios. Statistical significance was set at a *p*-value of 0.05. To assess whether there were any variations in the identification of *Alternaria* toxins across different food categories, the chi-square test was employed.

## 3. Results

### 3.1. Occurrence of Alternaria Toxins in the Zhejiang Province

#### 3.1.1. Contamination of Different Types of Food with *Alternaria* Toxins

The detection frequencies and concentrations of *Alternaria* toxins in various food samples collected from Zhejiang are presented in Table 1, along with their confidence intervals, which are shown in Table S2. Among the food items, wheat flour (113/116) had the highest detection rate, followed by Coix rice (27/34) and maize and its products (31/75). Fruits (4/100) had a lower detection rate of *Alternaria* toxins. The frequency of toxin detection in different food samples differed significantly (*p* < 0.05).

Overall, food samples were the most contaminated with TeA. Differences in the detection rates of TeA and TEN were not significant in the wheat flour samples (*p* > 0.05), where TeA concentrations averaged 36.2 µg/kg, with a maximum concentration of 183 µg/kg. The detection rate of TeA in maize and its products was significantly higher than that of the other three toxins (38.67%; *p* < 0.05), with a mean contamination concentration of 7.46 µg/kg. TeA was detected more frequently than other toxins in Coix rice (*p* < 0.05), with a relatively high average concentration of 34.73 µg/kg and the largest contamination concentration of 237 µg/kg, which was also the highest among all samples. Only TeA and TEN contaminations were detected in the fruit samples, with maximum contamination concentrations of 103 and 12.7 µg/kg, respectively.

#### 3.1.2. Co-Contamination with *Alternaria* Toxins

Detailed combinations of different *Alternaria* toxins that co-occurred in various food groups are shown in Table 2. In this study, 17.23% of the samples were contaminated with one toxin, and 36.62% were contaminated with two or more toxins. Six samples were contaminated with four toxins simultaneously, representing 1.85% of the total samples, including four wheat flour samples, one Coix rice sample, and one maize-product sample. Co-contamination with *Alternaria* toxins is common; TeA was the predominant contaminant in terms of both prevalence and concentration, with wheat flour being the most contaminated food group.

### 3.2. Survey on Food Consumption

Table 3 shows the daily consumption of the studied food items by the residents of Zhejiang Province. As the dietary preferences of different age groups may vary, to obtain a more accurate assessment of exposure, this study analyzed the consumption of the whole population while dividing the population into five age groups. Due to the absence of studies on Coix rice consumption among these age groups, we studied its overall consumption in the studied population, and the average body weight was calculated to be 60 kg.

### 3.3. Dietary Exposure Assessment

#### 3.3.1. Point Assessment of *Alternaria* Toxins in Zhejiang Province Residents

The dietary exposure to the four mycotoxins found in various food products are presented in Table 4. Wheat flour was the primary source of food exposure. The P95 exposure for AOH was 0.0038 and 0.0128 µg/kg b.w. for wheat flour and coix rice, respectively. The P95 exposure for AME was 0.0047 µg/kg b.w. for Coix rice consumption, which exceeded the TTC value of 0.0025 µg/kg b.w. for AOH and AME. Populations had a higher risk of exposure to AOH and AME than to TeA and TEN. In the exposure assessment of P95, the foods with the highest exposure to TeA and TEN were Coix rice and fruits, with exposures of 0.17 and 0.01 µg/kg, respectively.

Combining the total exposure of the four food items, the HQ value for the average AOH exposure was 122.44%, which indicated a risk of exposure; and the HQ values of 752.77% and 299.03% for AOH and AME, respectively, for the extreme scenarios, which indicated a risk of exposure for some of the population with a high level of consumption, while the HQ values for TeA and TEN were much less than 100%, which indicated a lower risk of exposure. The findings of the risk characterization showed that exposure to AOH and AME among residents in Zhejiang Province needs to be monitored, and the monitoring of AOH and AME in wheat flour and Coix rice needs to be strengthened.

#### 3.3.2. Point Assessment of *Alternaria* Toxins in Different Age Groups

Due to the distinct dietary patterns of the different age groups, consumption and dietary exposure of these toxins were grouped by age in this study. Table 5 and Table 6 show the results of the dietary exposure assessment for different age groups exposed to AOH and AME, and TeA and TEN, respectively, in Zhejiang Province.

As shown in Table 5, most of the population in Zhejiang Province was exposed to acceptable levels of *Alternaria* toxins. In extreme cases, children may have some risk of exposure. Dietary exposures from consumption of wheat flour AOH were below the TTC value in both scenarios 1 and 2; however, in scenario 3, exposures exceeded the TTC value of 0.0025 µg/kg b.w. for all age groups, suggesting a potential health risk. Exposure to AOH in maize and its products was low, with exposures for all age groups below the TTC value in scenario 3, with children aged ≤ 6 years at 0.0013 (<0.0001–0.0025) µg/kg b.w. having the highest exposure. In scenarios 1 and 2, AOH exposure from fruit intake were below the TTC value for all age groups, and was within the acceptable limit; however, in scenario 3, the exposure for children aged ≤ 6 and 7–12 years were 0.0034 (<0.0001–0.0067) and 0.0025 (<0.0001–0.0051) µg/kg b.w., suggesting some risk of exposure for these two age groups in extreme cases of P95 pollutant concentration and P95 consumption.

The results of the AME risk assessment showed that the three scenarios of consumption of maize, its products, and fruits by all age groups suggested a low risk of exposure. The AME exposure of children aged ≤ 6 years and 7–12 years through ingestion of wheat flour in scenario 3 was 0.0028 (0.0028–0.0028) and 0.0027 (0.0027–0.0027) µg/kg b.w., which were higher than the TTC values, suggesting a certain exposure risk, while the rest of the age groups and scenarios suggested a low exposure risk.

The TTC value of 1.5 μg/kg b.w. was adopted for TeA and TEN. In the present study, wheat flour, maize, and its products, and fruits had much lower exposures than TTC. In Table 6, the highest exposure to TeA in children aged ≤ 6 years consuming P95 from wheat flour was 0.13 μg/kg b.w., which was only 8.67% of the TTC. Dietary exposure to TEN was all lower than or equal to 0.01 μg/kg b.w.

#### 3.3.3. Probabilistic Assessment of AOH Exposure Through Wheat Flour

As the assessment results in scenario 3 in Table 5 indicate that human wheat flour consumption exposure is above the TTC value of 0.0025 µg/kg b.w. for all age groups, probabilistic assessments have been added to provide a more accurate assessment of the results. As shown in Table 7, the results showed that dietary exposure levels varied across age groups at different percentiles and that, similar to the results of the point assessment, the risk of exposure was higher in children than in adults and elderly individuals. Exposure was 0.0025 (0.0022–0.0030) μg/kg b.w. for P92 levels in children aged ≤ 6 years and 0.0025 (0.0021–0.0029) for P93 levels in children aged 7–12 years. Exposure to P95 levels was 0.0025 (0.0020–0.0030) and 0.0025 (0.0024–0.0027) μg/kg b.w. for those aged 13–17 and 18–59 years, respectively, and 0.0024 (0.0022–0.0027) for those aged ≥60 years, showing a trend of decreasing exposure levels with age.

## 4. Discussion

This research has several limitations. Firstly, the types of foods collected were limited. In the absence of a more comprehensive survey on consumption patterns among residents of Zhejiang Province, this study identifies wheat as the primary source of toxins. Second, there was temporal heterogeneity in the samples; the consumption data utilized in the present study were collected between 2015 and 2017, whereas the samples were collected in 2019 and 2022. Furthermore, we could not consider the impact of seasonal variations. Finally, *Alternaria* toxins may have synergistic effects, and modified sympatric mycotoxins were not considered; therefore, their hazards may have been underestimated.

In this study, we examined the contamination concentrations of four *Alternaria* toxins (AOH, AME, TeA, and TEN) in four food items to assess the population’s dietary exposure in relation to the consumption by residents of Zhejiang Province. Owing to the lack of specified limit values for *Alternaria* toxins in food, the rate of exceedance in food could not be assessed. The results of dietary exposure assessment showed that children aged ≤ 6 and 7–12 years had a higher exposure risk than other age groups, wheat flour is the main source of exposure, and the exposure risk of AOH and AME was greater than that of TEN and TeA.

Among the four food items, wheat flour had the highest detection rates for *Alternaria* toxins at 97.41%, and TeA and TEN had detection rates of 90.52% and 91.38%, respectively. In another study, the detection rates of AME, AOH, TeA, and TEN in Chinese wheat grain were 67%, 62.3%, 100%, and 99.4%, respectively, with the highest concentration of TeA detected in 2034 μg/kg [31]. It is similar to our findings that the detection rates of TeA and TEN were higher than that of AOH and AME, and TeA caused the most contamination. A Russian wheat sample contained 15,000 μg/kg of TeA [54]. The results of available studies indicate that TeA is the main *Alternaria* toxin that contaminates wheat flour [32,55], probably due to the fact that the *Alternaria* strains responsible for wheat black spot produce the highest concentration of TeA among the various toxins [56]. Some studies have found that crops previously grown as maize and winter wheat, combined with minimum tillage, wet weather, and heavy precipitation, can lead to relatively high levels of toxins in wheat [32,57]. Due to the long growth cycle of wheat, it is susceptible to the influence of the climate. For example, persistent cloudy and rainy weather during the flowering period results in high humidity in the field, meaning that *Alternaria* species can easily infest wheat and accumulate toxins [58]; however, there was no significant correlation between commodity packaging and toxin content [59].

The detection rates of Coix rice in Zhejiang Province for AOH and AME were 5.88% (maximum contamination 48.8 μg/kg) and 17.65% (48.4 μg/kg), respectively, which were higher than those in Fujian Province (5% [7.96 μg/kg] and 10% [5.26 μg/kg], respectively) [50]. The detection rate of *Alternaria* toxins in maize and its products was 41.33% in this study, and the maximum contamination concentration of TeA was 105 µg/kg. Another study in China showed a detection rate of 43.3% of *Alternaria* toxins in maize grits, while TEN was not detected, and the maximum pollution concentration of TeA was 11 μg/kg [42]. The type of grain, temperature, humidity during the growth phase, and post-harvest storage conditions affect mycotoxin levels in grain species [11,51,54].

In the 100 fruit samples collected from Zhejiang Province, TeA and TEN were detected at a rate of 2%, and AOH and AME were not detected. AME and TeA were detected in 5% and 10% of 20 fresh fruit samples from Korea, respectively [44]. In Zhejiang Province, the detection rate of fresh fruits in Zhejiang Province was lower than that in Korea, which may be due to the different detection standards and fruits tested. Further, the lower detection of *Alternaria* toxins in fruit samples may be due to the fact that fresh fruits are not easily contaminated by *Alternaria* species because of their strict storage conditions and short storage time. Studies on fresh fruits are limited, and *Alternaria* toxins in fruit products have been studied in several countries and regions. A Swiss study found that AOH, AME, and TEN were not detected in whole tomato samples. However, different levels of contamination were observed in tomato products [60]. Similar findings were reported in a Chinese study [61], possibly owing to increased concentrations of toxins from processes such as dehydration and concentration [10,30,61]. Fruits are susceptible to contamination by *Alternaria* toxins due to their high water and nutrient content and fragile skin [2,10,30,47], and the use of contaminated raw materials may be one of the reasons for the contamination of fruit products.

Exposure assessment results showed that dietary exposure to AOH and AME was within acceptable limits for most of the population; however, children had a higher risk of exposure because of their lower body weight. The assessment results grouped by age indicated a tendency for exposure to decrease with age. An assessment of exposure to AME and AOH through tomato and its products among Chinese residents was 0.017 and 0.046 μg/kg b.w. in adults and 0.037 and 0.100 μg/kg b.w. in children, respectively [30]. Children are more than twice as exposed as adults. In another study in China, children aged 4–7 years had higher exposure to AOH and AME from wheat grains than the rest of the age group, with exposure rates of 0.007 and 0.004 µg/kg b.w., respectively [31]. Another study showed that children aged 2–7 and 8–12 years consumed cereal AME and AOH at exposures above the TTC values, and the maximum exposure was 0.0037 and 0.0171 µg/kg b.w., with exposures 1–2 times higher than those of adults [42]. The results of existing studies have shown that the exposure risk of children in some studies was higher than the TTC values. Children in their early growth and developmental stages are more susceptible to mycotoxins. More attention should be paid to the assessment of the exposure risk in the child population and the setting of limit values for *Alternaria* toxins in children’s foods as soon as possible.

Depending on the food group, residents of Zhejiang Province had greater exposure to Coix rice and wheat flour than to maize and its products and fruits. The lower exposure to maize compared with the other two cereals may be due to its lower consumption [62]. Some studies have found that exposure to *Alternaria* toxins is 2–4 times higher in cereal products than in fruits and their products [42]. The EFSA assessment also indicates that cereals and their products are the main source of dietary intake of these toxins [3], which may be due to the higher consumption of cereals than fruits. There is currently a need for increased regulation and risk assessment of *Alternaria* toxins in cereals and their products.

Point assessment is uncertain and has a limited assessment scope. Probabilistic assessments provide a range of results and more complete information on risk distribution, which improves the scientific validity and accuracy of the assessment results [10,62,63]. Fewer results on the probabilistic assessment of *Alternaria* toxins are available. The P90 exposure by wheat flour AOH was 0.0788 and 0.0688 μg/kg b.w. for boys and girls aged 7–10 years, respectively, in Shanghai [64], probably due to high sample contamination (6.6 μg/kg), which was higher than the P91 exposure of 0.0019 μg/kg b.w. for children aged 7–12 years in Zhejiang Province.

Most countries, including China, have not established limiting values for *Alternaria* toxins in food. The European Union has established the maximum permitted concentrations of AOH, AME, and TeA in processed agricultural products and foods for infants and young children [65]. In risk assessment studies, the exposure assessment of *Alternaria* toxins focused on AOH and AME, probably because of the higher risk of these two toxins at exposures above the TTC value. According to our results and those of other studies, exposure to TeA was the highest, but was lower than the TTC value, and the assessment results showed a low exposure risk. The TTC value was established based on genotoxicity [2], while TeA has strong acute toxicity, and there is a lack of toxicological data related to chronic toxicity and reproductive developmental toxicity. The effects of TEN on humans need to be further investigated. Based on the available results, TEN exposure is much lower than the TTC values. Several studies have identified synergistic effects of concomitant exposure to a variety of *Alternaria* toxins. For example, synergistic toxic effects have been demonstrated in colonic cells with concomitant exposure to AOH and AME [66]. Further, both AME-TEN and TeA-TEN combinations showed almost synergistic toxic effects on liver cells [67]. AOH at 100 mg/kg had fetotoxic effects in mice, while AME at 50 mg/kg produced no effect, but the combination of AOH and AME at 25 mg/kg produced a significant response, indicating a synergistic effect [24]. However, we were not able to identify any studies conducting a risk assessment of the synergistic effects of *Alternaria* toxins, and the results of existing studies include only preliminary risk assessments; thus, verifying their results requires further research and the establishment of a comprehensive regulatory system and safety regulations as soon as possible for a more accurate risk assessment of *Alternaria* toxins.

## 5. Conclusions

The study showed that the contamination levels of *Alternaria* toxins in fruits were lower than those in cereals and cereal products. TeA was the most common toxin, and its contamination concentration was higher than that of the other three toxins. According to the findings of the population-wide dietary exposure assessment, consuming wheat flour and Coix rice put people in Zhejiang Province at risk of exposure to AOH and AME. Further assessment by age group showed that in extreme cases, all age groups were at potential risk of AOH exposure through wheat flour consumption, while children aged ≤6 and 7–12 years were at risk of AME exposure. This suggests that wheat flour is the main source of exposure to *Alternaria* toxins in Zhejiang Province, and that children are at a higher risk of exposure than adults due to their lower body weight. Exposure to both TeA and TEN was within acceptable limits. In view of the above, we should set the limits for *Alternaria* toxins in various types of food as soon as possible, strengthen our regulatory efforts, and actively conduct relevant studies to explore effective ways to reduce toxin contamination in food to safeguard public health.

## Figures and Tables

**Table 1:** Contamination levels of *Alternaria* toxins in foods marketed in Zhejiang province, China (*n* = 325).

Mycotoxin	Food Name	Detection Rate (%)	Mean Pollution Concentration MB ^5^ (LB ^6^-UB ^7^) (µg/kg)	P50 Pollution Concentration MB (LB-UB) (µg/kg)	P95 Pollution Concentration MB (LB-UB) (µg/kg)	Max Pollution Concentration (µg/kg)
AOH ^1^	Wheat flour	(6/116)	2.87	1.50	4.30	65.10
		5.17	(1.44–4.29)	(0–3)	(3.18–5.43)	
	Maize and its products	(2/75)	2.11	1.50	1.50	27.00
		2.67	(0.65–3.57)	(0–3.0)	(0–3.0)	
	Coix rice	(2/34)	3.49	1.50	8.64	48.80
		5.88	(2.08–4.90)	(0–3.0)	(7.67–9.62)	
	Fruits	(0/100)	1.50	1.50	1.50	0
		0	(0–3.0)	(0–3.0)	(0–3.0)	
AME ^2^	Wheat flour	(28/116)	1.74	0.40	2.10	63.40
		24.14	(1.43–2.04)	(0–0.80)	(2.10–2.10)	
	Maize and its products	(7/75)	0.73	0.40	1.0	20.30
		9.33	(0.37–1.09)	(0–0.80)	(1.0–1.0)	
	Coix rice	(6/34)	2.06	0.40	3.21	48.40
		17.65	(1.73–2.39)	(0–0.80)	(3.21–3.21)	
	Fruits	(0/100)	0.40	0.40	0.40	0
		0	(0–0.80)	(0–0.80)	(0–0.80)	
TeA ^3^	Wheat flour	(105/116)	36.20	28.80	95.38	183.00
		90.52	(36.13–36.27)	(28.80–28.80)	(95.38–95.38)	
	Maize and its products	(29/75)	7.46	0.75	35.71	105.00
		38.67	(7.00–7.92)	(0–1.5)	(35.71–35.71)	
	Coix rice	(25/34)	34.73	11.95	111.67	237.00
		73.53	(34.54–34.93)	(11.95–11.95)	(111.67–111.67)	
	Fruits	(2/100)	1.94	0.75	0.75	103.00
		2.00	(1.20–2.67)	(0–1.50)	(0–1.50)	
TEN ^4^	Wheat flour	(106/116)	3.03	2.00	9.15	17.60
		91.38	(3.02–3.05)	(2.00–2.00)	(9.15–9.15)	
	Maize and its products	(3/75)	0.25	0.15	0.15	7.10
		4.00	(0.11–0.40)	(0–0.30)	(0–0.30)	
	Coix rice	(13/34)	0.57	0.15	1.35	8.60
		38.24	(0.48–0.67)	(0–0.30)	(1.35–1.35)	
	Fruits	(2/100)	0.36	0.15	0.15	12.70
		2	(0.22–0.51)	(0–0.30)	(0–0.30)	

^1^ AOH, alternariol; ^2^ AME, alternariol monomethyl ether; ^3^ TeA, tenuazonic acid; ^4^ TEN, tentoxin; ^5^ MB, medium bound, ND = 1/2 LOD; ^6^ LB, lower bound, ND = 0; ^7^ UB, upper bound, ND = LOD.

**Table 2:** Combinations of *Alternaria* toxins in food.

Co-Occurrence	Combination	Frequency
2 toxins	AOH-TeA	1/325 (0.31%)
	AME-TeA	6/325 (1.85%)
	AME-TEN	1/325 (0.31%)
	TeA-TEN	75/325 (23.08%)
	Total	83/325 (25.54%)
3 toxins	AME-TeA-TEN	28/325 (8.62%)
	TeA-TEN-AOH	2/325 (0.62%)
	Total	30/325 (9.23%)
4 toxins	AOH-AME-TeA-TEN	6/325 (1.85%)
	Total	6/325 (1.85%)

**Table 3:** Daily consumption of the samples by the participants.

Age Group	Food Name	Consumption (g/Day)		
		Mean	P50	P95
≤6 years	Maize and its products	5.16	2.00	16.51
	Wheat flour	6.69	2.86	25.71
	Fruits	10.91	3.33	45.00
7–12 years	Maize and its products	6.91	2.67	21.43
	Wheat flour	10.05	3.33	42.86
	Fruits	13.59	4.11	57.14
13–17 years	Maize and its products	8.65	2.67	28.57
	Wheat flour	11.26	3.67	42.86
	Fruits	13.95	5.00	57.14
18–59 years	Maize and its products	7.33	3.00	25.71
	Wheat flour	14.22	5.00	57.14
	Fruits	14.67	4.87	60.00
≥60 years	Maize and its products	8.59	3.00	28.57
	Wheat flour	13.01	5.00	45.43
	Fruits	13.63	4.00	60.00
All	Maize and its products	7.45	3.86	25.71
	Wheat flour	13.20	5.00	50.00
	Fruits	14.16	4.33	57.14
	Coix rice	29.82	20.00	88.85

**Table 4:** Exposure to *Alternaria* toxins among residents of Zhejiang Province.

Foodstuffs	PDI ^1^ (µg/kg bw/Day)											
	AOH			AME			TeA			TEN		
	Scenario 1 ^2^	Scenario 2 ^3^	Scenario 3 ^4^	Scenario 1	Scenario 2	Scenario 3	Scenario 1	Scenario 2	Scenario 3	Scenario 1	Scenario 2	Scenario 3
Maize and its products	0.0003	0.0001	0.0007	0.0001	<0.0001	0.0005	<0.01	<0.01	0.02	<0.01	<0.01	<0.01
Wheat flour	0.0007	0.0001	**0.0038**	0.0004	<0.0001	0.0019	0.01	<0.01	0.09	<0.01	<0.01	0.01
Fruits	0.0004	0.0001	0.0015	0.0001	<0.0001	0.0004	<0.01	<0.01	<0.01	<0.01	<0.01	<0.01
Coix rice	0.0017	0.0005	**0.0128**	0.0010	0.0001	**0.0047**	0.02	<0.01	0.17	<0.01	<0.01	<0.01
Total	0.0031	0.0009	0.0188	0.0016	0.0002	0.0075	0.03	0.01	0.27	<0.01	<0.01	0.01
HQ (%) ^5^	122.44	34.02	752.77	65.19	9.07	299.03	1.82	0.44	17.83	0.07	0.02	0.69

^1^ PDI, probable daily intake; ^2^ Scenario 1, mean contamination level (MB) combined with the mean daily consumption; ^3^ Scenario 2, P50 contamination levels (MB) combined with the P50 daily consumption. ^4^ Scenario 3, P95 contamination levels (MB) combined with the P95 daily consumption; ^5^ HQ, the hazard quotient. Bold font indicates some risk of exposure. If HQ is < 100%, the health risk is considered acceptable; if HQ is >100%, the health risk is considered unacceptable.

**Table 5:** Dietary intake of AOH and AME in different age groups for the three-point assessment models.

Toxins	Age Group	PDI ^1^ MB ^2^ (LB ^3^–UB ^4^) (µg/kg bw/Day)								
		Wheat Flour			Maize and Its Products			Fruits		
		Scenario 1 ^5^	Scenario 2 ^6^	Scenario 3 ^7^	Scenario 1	Scenario 2	Scenario 3	Scenario 1	Scenario 2	Scenario 3
AOH	≤6	0.0010	0.0002	0.0058	0.0006	0.0002	0.0013	0.0008	0.0002	0.0034
		(0.0005–0.0015)	(<0.0001–0.0005)	(0.0043–0.0074)	(0.0002–0.0009)	(<0.0001–0.0003)	(<0.0001–0.0025)	(<0.0001–0.0016)	(<0.0001–0.0005)	(<0.0001–0.0067)
	7–12	0.0009	0.0001	0.0055	0.0004	0.0001	0.0009	0.0006	0.0002	0.0025
		(0.0004–0.0013)	(<0.0001–0.0003)	(0.0041–0.0070)	(0.0001–0.0007)	(<0.0001–0.0002)	(<0.0001–0.0019)	(<0.0001–0.0012)	(<0.0001–0.0004)	(<0.0001–0.0051)
	13–17	0.0006	0.0001	0.0036	0.0003	0.0001	0.0008	0.0004	0.0001	0.0016
		(0.0003–0.0010)	(<0.0001–0.0002)	(0.0027–0.0046)	(0.0001–0.0006)	(<0.0001–0.0002)	(<0.0001–0.0016)	(<0.0001–0.0008)	(<0.0001–0.0003)	(<0.0001–0.0033)
	18–59	0.0007	0.0001	0.0040	0.0003	0.0001	0.0006	0.0004	0.0001	0.0015
		(0.0003–0.0010)	(<0.0001–0.0002)	(0.0030–0.0051)	(0.0001–0.0004)	(<0.0001–0.0001)	(<0.0001–0.0013)	(<0.0001–0.0007)	(<0.0001–0.0002)	(<0.0001–0.0029)
	≥60	0.0006	0.0001	0.0033	0.0003	0.0001	0.0007	0.0003	0.0001	0.0015
		(0.0003–0.0009)	(<0.0001–0.0003)	(0.0025–0.0042)	(0.0001–0.0005)	(<0.0001–0.0002)	(<0.0001–0.0014)	(<0.0001–0.0007)	(<0.0001–0.0002)	(<0.0001–0.0030)
AME	≤6	0.0006	0.0001	0.0028	0.0002	<0.0001	0.0008	0.0002	0.0001	0.0009
		(0.0005–0.0007)	(<0.0001–0.0001)	(0.0028–0.0028)	(0.0001–0.0003)	(<0.0001–0.0001)	(0.0008–0.0008)	(<0.0001–0.0004)	(<0.0001–0.0001)	(<0.0001–0.0018)
	7–12	0.0005	<0.0001	0.0027	0.0001	<0.0001	0.0006	0.0002	<0.0001	0.0007
		(0.0004–0.0006)	(<0.0001–0.0001)	(0.0027–0.0027)	(0.0001–0.0002)	(<0.0001–0.0001)	(0.0006–0.0006)	(<0.0001–0.0003)	(<0.0001–0.0001)	(<0.0001–0.0014)
	13–17	0.0004	<0.0001	0.0018	0.0001	<0.0001	0.0005	0.0001	<0.0001	0.0004
		(0.0003–0.0005)	(<0.0001–0.0001)	(0.0018–0.0018)	(0.0001–0.0002)	(<0.0001–<0.0001)	(0.0005–0.0005)	(<0.0001–0.0002)	(< 0.0001–0.0001)	(<0.0001–0.0009)
	18–59	0.0004	<0.0001	0.0020	0.0001	<0.0001	0.0004	0.0001	<0.0001	0.0004
		(0.0003–0.0005)	(<0.0001–0.0001)	(0.0020–0.0020)	(<0.0001–0.0001)	(<0.0001–<0.0001)	(0.0004–0.0004)	(<0.0001–0.0002)	(<0.0001–0.0001)	(<0.0001–0.0008)
	≥60	0.0001	<0.0001	0.0016	0.0001	<0.0001	0.0005	0.0001	<0.0001	0.0004
		(0.0001–0.0002)	(<0.0001–0.0001)	(0.0016–0.0016)	(0.0001–0.0002)	(<0.0001–<0.0001)	(0.0005–0.0005)	(<0.0001–0.0002)	(< 0.0001–0.0001)	(<0.0001–0.0008)

^1^ PDI: probable daily intake. ^2^ MB, medium bound, ND = 1/2 LOD; ^3^ LB, lower bound, ND = 0; ^4^ UB, upper bound, ND = LOD. ^5^ Scenario 1: Mean contamination levels in the samples combined with the mean daily consumption. ^6^ Scenario 2: P50 contamination levels in the samples combined with the P50 daily consumption. ^7^ Scenario 3: P95 contamination levels in the samples combined with the P95 daily consumption. All scenarios use the average body weight.

**Table 6:** Dietary intake of TeA and TEN for different age groups.

Toxins	Age Group	PDI ^1^ MB ^2^ (LB ^3^–UB ^4^) (µg/kg bw/Day)								
		Wheat Flour			Maize and Its Products			Fruits		
		Scenario 1 ^5^	Scenario 2 ^6^	Scenario 3 ^7^	Scenario 1	Scenario 2	Scenario 3	Scenario 1	Scenario 2	Scenario 3
TeA	≤6	0.01	<0.01	0.13	<0.01	<0.01	0.03	<0.01	<0.01	<0.01
		(0.01–0.01)	(<0.01–<0.01)	(0.13–0.13)	(<0.01–<0.01)	(<0.01–<0.01)	(0.03–0.03)	(<0.01–<0.01)	(<0.01–<0.01)	(<0.01–<0.01)
	7–12	0.01	<0.01	0.12	<0.01	<0.01	0.02	<0.01	<0.01	<0.01
		(0.01–0.01)	(<0.01–<0.01)	(0.12–0.12)	(<0.01–<0.01)	(<0.01–<0.01)	(0.02–0.02)	(<0.01–<0.01)	(<0.01–<0.01)	(<0.01–<0.01)
	13–17	0.01	<0.01	0.08	<0.01	<0.01	0.02	<0.01	<0.01	<0.01
		(0.01–0.01)	(<0.01–<0.01)	(0.08–0.08)	(<0.01–<0.01)	(<0.01–<0.01)	(0.02–0.02)	(<0.01–<0.01)	(<0.01–<0.01)	(<0.01–<0.01)
	18–59	0.01	<0.01	0.09	<0.01	<0.01	0.01	<0.01	<0.01	<0.01
		(0.01–0.01)	(<0.01–<0.01)	(0.09–0.09)	(<0.01–<0.01)	(<0.01–<0.01)	(0.01–0.01)	(<0.01–<0.01)	(<0.01–<0.01)	(<0.01–<0.01)
	≥60	<0.01	<0.01	0.07	<0.01	<0.01	0.02	<0.01	<0.01	<0.01
		(<0.01–<0.01)	(<0.01–<0.01)	(0.07–0.07)	(<0.01–<0.01)	(<0.01–<0.01)	(0.02–0.02)	(<0.01–<0.01)	(<0.01–<0.01)	(<0.01–<0.01)
TEN	≤6	<0.01	<0.01	0.01	<0.01	<0.01	<0.01	<0.01	<0.01	<0.01
		(<0.01–<0.01)	(<0.01–<0.01)	(0.01–0.01)	(<0.01–<0.01)	(<0.01–<0.01)	(<0.01–<0.01)	(<0.01–<0.01)	(<0.01–<0.01)	(<0.01–<0.01)
	7–12	<0.01	<0.01	0.01	<0.01	<0.01	<0.01	<0.01	<0.01	<0.01
		(<0.01–<0.01)	(<0.01–<0.01)	(0.01–0.01)	(<0.01–<0.01)	(<0.01–<0.01)	(<0.01–<0.01)	(<0.01–<0.01)	(<0.01–<0.01)	(<0.01–<0.01)
	13–17	<0.01	<0.01	0.01	<0.01	<0.01	<0.01	<0.01	<0.01	<0.01
		(<0.01–<0.01)	(<0.01–<0.01)	(0.01–0.01)	(<0.01–<0.01)	(<0.01–<0.01)	(<0.01–<0.01)	(<0.01–<0.01)	(<0.01–<0.01)	(<0.01–<0.01)
	18–59	<0.01	<0.01	0.01	<0.01	<0.01	<0.01	<0.01	<0.01	<0.01
		(<0.01–<0.01)	(<0.01–<0.01)	(0.01–0.01)	(<0.01–<0.01)	(<0.01–<0.01)	(<0.01–<0.01)	(<0.01–<0.01)	(<0.01–<0.01)	(<0.01–<0.01)
	≥60	<0.01	<0.01	0.01	<0.01	<0.01	<0.01	<0.01	<0.01	<0.01
		(<0.01–<0.01)	(<0.01–<0.01)	(0.01–0.01)	(<0.01–<0.01)	(<0.01–<0.01)	(<0.01–<0.01)	(<0.01–<0.01)	(<0.01–<0.01)	(<0.01–<0.01)

^1^ PDI: probable daily intake. ^2^ MB, medium bound, ND = 1/2 LOD; ^3^ LB, lower bound, ND = 0; ^4^ UB, upper bound, ND = LOD. ^5^ Scenario 1: Mean contamination levels in the samples combined with the mean daily consumption. ^6^ Scenario 2: P50 contamination levels in the samples combined with the P50 daily consumption. ^7^ Scenario 3: P95 contamination levels in the samples combined with the P95 daily consumption. All scenarios use the average body weight.

**Table 7:** Probabilistic assessment of dietary intake of AOH through wheat flour in different age groups.

Age Group	Percentile of AOH Estimates Through Wheat Flour Exposure and 90% Confidence Limits (µg/kg bw/Day)					
	P91 ^1^	P92	P93	P94	P95	P96
≤6	0.0022 ^2^	0.0025	0.0029	0.0034	0.0040	0.0049
	(0.0019–0.0026) ^3^	(0.0022–0.0030)	(0.0025–0.0034)	(0.0029–0.0040)	(0.0034–0.0047)	(0.0041–0.0057)
7–12	0.0019	0.0022	0.0025	0.0029	0.0035	0.0042
	(0.0016–0.0022)	(0.0018–0.0025)	(0.0021–0.0029)	(0.0025–0.0034)	(0.0030–0.0040)	(0.0036–0.0050)
13–17	0.0014	0.0015	0.0018	0.0021	0.0025	0.0030
	(0.0011–0.0016)	(0.0012–0.0018)	(0.0014–0.0021)	(0.0017–0.0025)	(0.0020–0.0030)	(0.0025–0.0037)
18–59	0.0014	0.0016	0.0018	0.0021	0.0025	0.0031
	(0.0013–0.0014)	(0.0015–0.0016)	(0.0017–0.0019)	(0.0020–0.0022)	(0.0024–0.0027)	(0.0030–0.0033)
≥60	0.0014	0.0015	0.0018	0.0021	0.0024	0.0030
	(0.0012–0.0015)	(0.0014–0.0017)	(0.0016–0.0019)	(0.0019–0.0023)	(0.0022–0.0027)	(0.0027–0.0033)

^1^ Indicates the percentile of the probability distribution of AOH intake. ^2^ Indicates mean value of exposure. ^3^ Represents a 90% confidence interval.

## Data Availability

The data in this study are sensitive and involve certain personal privacy and commercial brand information. To obtain the raw data, please contact the corresponding author.

## Supplementary Materials

The following supporting information can be downloaded at: https://www.mdpi.com/article/10.3390/foods14193298/s1, Table S1: LOD and LOQ for *Alternaria* toxins testing; Table S2: Confidence 95% intervals for detection and concentration of *Alternaria* toxins in foods.
