# Supplementary material for: Prevalence and Exposure Assessment of Alternaria Toxins in Zhejiang Province, China
Source: Foods. 2025 Sep 23;14(19):3298. doi: 10.3390/foods14193298 (PMC12523592; doi:10.3390/foods14193298)
Supplement: Supplementary file 1 [file foods-14-03298-s001.zip › foods-3852752-supplementary.pdf]

Table S1. LOD and LOQ for *Alternaria* toxins testing

Mycotoxin	LOD(μg/kg)	LOQ(μg/kg)
AOH	3	9
AME	0.8	2.4
TeA	1.5	4.5
TEN	0.3	0.9

Table S2. Confidence 95% intervals for detection and concentration of *Alternaria* toxins in foods.

Mycotoxin	Food Name	Confidence intervals for prevalence (%)	Confidence intervals for concentrations(μg/kg)
**AOH**	Wheat flour	3.11-7.23	1.57-4.17
	Maize and its products	0.81-4.53	1.27-2.95
	Coix rice	1.85-9.91	0.55-6.43
	Fruits	0	1.50-1.50
**AME**	Wheat flour	20.17-28.11	0.46-3.02
	Maize and its products	5.97-12.69	0.21-1.25
	Coix rice	11.11-24.19	0-4.83
	Fruits	0	0.40-0.40
**TeA**	Wheat flour	87.80-93.24	30.23-42.17
	Maize and its products	33.05-44.29	3.72-11.20
	Coix rice	68.16-82.90	17.69-51.77
	Fruits	0.60-3.40	0-3.97
**TEN**	Wheat flour	88.77-93.99	2.43-3.63
	Maize and its products	1.74-6.26	0.07-0.43
	Coix rice	29.91-46.57	0.08-1.06
	Fruits	0.60-3.40	0.06-0.66
